# Recovery of *Salmonella* bacterial isolates from pooled fecal samples from horses

**DOI:** 10.1111/jvim.16586

**Published:** 2022-11-25

**Authors:** Jose I. Goni, Kenitra Hendrix, Janice Kritchevsky

**Affiliations:** ^1^ Department of Veterinary Clinical Sciences College of Veterinary Medicine West Lafayette Indiana USA; ^2^ State of Indiana Animal Disease Diagnostic Laboratory West Lafayette Indiana USA

**Keywords:** culture, horse, pool, series

## Abstract

**Background:**

It is important to determine if a horse is shedding *Salmonella* spp., but a complete culture series can be cost prohibitive.

**Objectives:**

Determine the optimal pooling technique to maintain high sensitivity of *Salmonella* spp. culture using spiked samples, and then demonstrate the efficacy of this protocol on clinical submissions.

**Hypothesis:**

Pooled fecal samples are as sensitive as 5 individual cultures for the detection of *Salmonella* shedding.

**Animals:**

A single *Salmonella*‐negative horse from the university herd, and 19 hospitalized horses.

**Methods:**

*Salmonella*‐free fecal samples were spiked with different amounts of *Salmonella* spp. (10^2^, 10^3^, 10^4^, and 10^5^ colony forming units [cfu]) and homogenized to evaluate pooled samples. Five individual fecal samples were collected from 19 hospitalized horses. Ten‐gram aliquots of each individual sample were combined to make a pooled sample. Both individual and pooled samples were cultured for *Salmonella* spp. The identity of bacterial isolates was confirmed by matrix‐assisted laser desorption‐ionization time of flight mass spectrometry.

**Results:**

A 10^2^ cfu concentration of *Salmonella* spp. could be recovered from a spiked *Salmonella*‐free fecal sample. Homogenization protocols indicated that the addition of 20 mL of broth to the pooled sample improved recovery, whereas homogenization time did not. Of the 19 horses tested, 5 were positive for *Salmonella*. In all instances, *Salmonella* spp. were recovered from the fecal pool as well as individual samples.

**Conclusions and Clinical Importance:**

Pooling of 5 fecal samples for *Salmonella* culture is a sensitive and cost‐effective diagnostic approach to detect horses that are shedding the organism.

**Abbreviations**
CFU: colony‐forming unit
GI: gastrointestinal
PCR: polymerase chain reaction

## 1 INTRODUCTION

It is important to identify horses that are shedding *Salmonella* spp. in their feces because of zoonotic risks and biosecurity concerns. Doing so can be difficult because horses with *Salmonella* spp. infections are often asymptomatic or can be clinically indistinguishable from those with colitis caused by other pathogens. Additionally, horses may shed the organism intermittently. Previous studies^1^, ^2^ found that a single culture identified between 33% and 55% of horses that were later found to be positive when additional cultures were performed. Why *Salmonella* and other enteric pathogens are shed intermittently is not well understood. It is hypothesized to be the result of local immune and inflammatory factors, but the exact mechanisms have yet to be elucidated.^3^

Because of the intermittent nature of fecal shedding, the most widely accepted protocol for diagnosing *Salmonella* in horses is collecting 5 individual fecal samples for *Salmonella* culture.^2^ As the cost of laboratory testing increases, however, submitting 5 individual cultures has become cost prohibitive in many instances. This expense leads practitioners to submit fewer fecal cultures.

Pooled fecal cultures are a well‐established cost‐effective way to estimate the prevalence of *Salmonella* infection in cattle and swine herds.^4^, ^5^ They generally are performed by combining feces collected on a single day from multiple animals. Pooling samples has been described as a means to identify horses with *Salmonella* using PCR but not culture.^6^ Although PCR has an important role in the identification of *Salmonella* positive animals, PCR results do not always match fecal culture results.^7^ An animal that is intermittently shedding the organism might be misidentified as a nonshedder by a single PCR just as it can be misidentified by a single culture. Although PCR assays are improving in their agreement with bacterial cultures, PCR is still not as reliable as culture.^8^ Additionally, isolates obtained by culture are required for serotype determination.

Our goal was to determine if pooling 5 fecal samples collected at individual time points was as accurate as culturing 5 single samples to identify feces that contain *Salmonella* spp.

## 2 MATERIALS AND METHODS

The study protocol was approved by Purdue University's Institutional Animal Care and Use Committee.

*In vitro studies*: *Salmonella* Group E: *Salmonella* Group E was selected for the spiking studies because it is the most common isolate cultured in submissions from horses. An American Type Culture Collection *Salmonella* Group E (ATCC 9270) isolate was obtained and cultured in the Indiana Animal Disease Diagnostic Laboratory's bacteriology laboratory for use in the study according to established protocols for maintaining quality control isolates.

*Control fecal samples*: Fresh fecal samples were collected for 5 consecutive days from a horse housed on the Purdue College of Veterinary Medicine's property. Routine *Salmonella* culture was performed on 5 individual samples, and all were negative. This horse served as the *Salmonella‐*free fecal donor for the remainder of the study.

*Spiking and pooling of feces*: *Salmonella* was cultured in broth and the cfu/mL were determined by standard plate counts. Desired infective doses of 10^2^, 10^3^, 10^4^, and 10^5^ total cfu were obtained by serial dilution. These concentrations were selected because they correspond to what is frequently obtained in food animal pooled samples.^4^ Feces in 20‐g aliquots from the fecal donor horse were spiked with each desired concentration. Spiked samples were mixed using an electric homogenizer (Stomacher 400Circulator [Seward, UK]) for 1 minute at 230 rpm. Ten grams were cultured for *Salmonella* to show spiking was successful.

In phase 1 of the pooling study, the remaining 10 g were pooled with 40 g of *Salmonella*‐negative feces, replicating a pool of 1 positive field sample and 4 negative field samples. Fecal pools were homogenized for 1 minute at 230 rpm.

In phase 2 of the pooling study, 4 homogenization methods were tested on triplicate 50 g pools containing a single 10 g sample spiked with 10^2^ total cfu of *Salmonella* to determine the ideal homogenization protocol. Each pool then was divided for 5 individual cultures. The homogenization protocols tested were: 1 minute with no broth, 5 minutes with no broth, 1 minute with 20 mL broth, and 5 minutes with 20 mL broth. All pools were processed at 230 rpm. The broth used in the homogenization protocol was Nutrient Broth (BBL, BD Life Sciences). It is formulated with peptone and beef extract and serves as a general‐purpose broth (Table 1).

**TABLE 1:** Isolation of *Salmonella* from spiked pools of feces

		Result						
		Sample number						
50 g bag	Processing method	1	2	3	4	5	False negative per pool	False negative per method
1a	1 min; no broth	Positive	Positive	Negative	Positive	Negative	2	5
1b		Positive	Positive	Positive	Positive	Negative	1	
1c		Positive	Positive	Positive	Negative	Negative	2	
5a	5 min; no broth	Positive	Positive	Positive	Negative	Positive	1	7
5b		Positive	Negative	Negative	Negative	Negative	4	
5c		Negative	Negative	Positive	Positive	Positive	2	
B1a	1 min; 20 mL broth	Positive	Positive	Positive	Negative	Positive	1	1
B1b		Positive	Positive	Positive	Positive	Positive	0	
B1c		Positive	Positive	Positive	Positive	Positive	0	
B5a	5 min; 20 mL broth	Positive	Positive	Positive	Positive	Positive	0	1
B5b		Positive	Positive	Positive	Positive	Positive	0	
B5c		Positive	Positive	Positive	Positive	Negative	1	

*Salmonella* culture: Ten grams of feces, whether from an individual sample or a pooled sample, were added to 100 mL of tetrathionate broth and incubated 48 hours at 36 ± 2°C. The broth then was used to inoculate brilliant green and XLT‐4 agar plates which were incubated up to 48 hours at 36 ± 2°C. Colonies characteristic of *Salmonella* spp. were identified by matrix‐assisted laser desorption‐ionization time of flight mass spectrometry (MALDI‐TOF) using the direct transfer method and a minimum score of 1.8 for genus identification using the Biotyper software version current at the time of identification (Bruker Scientific Corp., Billerica, Massachusetts).

*Study population*: Horses included in the study were presented to the teaching hospital between September 2018 and April 2021. Study eligibility was restricted to those horses for which clinicians elected to submit 5 individual samples for *Salmonella* culture either as a screen or because they were classified as high risk. Horses that exhibited clinical signs consistent with gastrointestinal disease such as diarrhea or colic, or horses with fever or leukopenia were cultured most frequently. Signalment, presenting complaint, days spent in the hospital, age, sex, breed, outcome, and culture results (individual samples and pooled sample) were obtained from the horse's medical record (see Table S1).

*Sample handling*: Pools were created from 10 g of each of the 5 samples. Fecal sampling consisted of 1 to 3 fecal samples collected daily until 5 samples were obtained (5‐series fecal sample). More than 1 sample was collected per day if the clinician believed the horse would be discharged before 5 days. At least 8 hours elapsed between collection of individual samples. Fecal samples were collected from fresh manure off of the stall floor before cleaning. All samples were submitted in their individual containers to the laboratory for bacteriology culture and identification of *Salmonella* spp. The pooled sample was made up of 10‐g aliquots from each individual sample to make a 50‐g submission. Samples collected outside of regular business hours were refrigerated at 4°C before submission. During weekdays, individual samples were refrigerated before submission for a maximum of 24 hours. During weekends, individual samples were kept ≤60 hours before submission. Pooled samples were formed as the aliquots of the individual samples were added. Thus, the longest the first part of a pooled sample was refrigerated before submission was 120 hours. Horses were not included if their feces were too liquid to measure accurately or if <10 g were collected. No fecal or rectal swabs were included.

After the initial results were evaluated, the records of all horses that had fecal cultures for *Salmonella* isolation were examined. These represented horses that had a single or series of *Salmonella* cultures submitted even though there was no corresponding pooled sample. This approach was used to determine the frequency of mixed culture results (at least 1 of the 5 positives for *Salmonella* spp. and at least 1 with no *Salmonella* spp. detected). A total of 64 horses fit these criteria during the study period from September 2018 to April 2021. Descriptive statistics were reported using median and range.

## 3 RESULTS

Phase 1 of the pooling protocol indicated that S*almonella* could be recovered successfully from an individual sample spiked with 10^2^ cfu. However, the sample spiked with 10^3^ cfu was negative on culture, which led us to pursue a comparison of homogenization protocols in Phase 2. Phase 2 of the pooling study indicated that the addition of 20 mL of broth to the pooled sample improved the homogenization protocol. With 15 cultures representing each of 4 homogenization protocols, only 1 false negative resulted from each protocol using broth. No difference was found in the outcome of homogenizing for 1 minute versus 5 minutes. However, the pooling protocols that did not include the addition of broth resulted in 5 and 7 false negative samples, using 1‐ and 5‐minute processing times, respectively.

Based on phase 2 results, the pooling protocol applied to the clinical portion of the study using patient feces as samples was to pool 5 10‐g samples, add 20 mL nutrient broth, and process in the homogenizer at 230 rpm for 1 minute.

Individual and pooled fecal samples were collected from 19 horses. Demographics of the study population are given in Table S1. The median age was 8 years (range, 1‐21 years). The median number of days of hospitalization was 9 days (range, 2‐160 days). The most common presenting complaint was colic (n = 8, 42.1%), followed by diarrhea (n = 4, 21%), colitis (n = 2, 10.5%) and fever (n = 2, 10.5%). Two of the 5 horses presented with both diarrhea and fever. Horses with ophthalmic disorders (n = 2; 10.5%) were sampled because they shared barns or caretakers with horses that tested positive for *Salmonella* spp. at some point during their hospitalization. One horse was euthanized during hospitalization. The remainder were discharged from the hospital (n = 18, 94.7%).

### 3.1 Fecal culture results

The results of the 5‐series culture samples and the corresponding pool sample agreed in all cases. Five horses tested positive for their 5‐series culture samples and their corresponding pool sample (n = 5; 26.3% of the total). In all 5 horses, all of the submitted individual cultures were positive for *Salmonella* species. Information about the result of the fecal culture for each horse is given in Table S2.

Among the horses that tested positive for *Salmonella* spp., 4 (80%) presented initially with clinical signs related to gastrointestinal problems such as colic or diarrhea, and 1 presented initially for an ophthalmic disorder (eye trauma). All horses that tested positive for *Salmonella* spp. survived their hospitalization and were discharged from the clinic.

To determine how common intermittent shedding was in our population, the records of all horses that had at least 1 *Salmonella* culture submitted during the study period were evaluated. This group consisted of 64 horses. Of those, 11 had at least 1 positive *Salmonella* culture from 12 submissions. One of the horses was tested 3 times to check for presence of *Salmonella* spp. The final set of 5 samples was negative. The results of each horse and culture are given in Table S3. Of those submissions, 6 were positive on all 5 cultures, 4 on 2 cultures, and 2 on 1 culture. When combining the horses with pooled culture with those that were not pooled, 83 horses had fecal cultures submitted. Of those, 16 had at least 1 positive culture identified. In 10 of those instances, all samples were positive whereas in 6 mixed results (positive for *Salmonella* and no *Salmonella* detected) were obtained.

## 4 DISCUSSION

All horses the pooled samples of which tested positive for *Salmonella* spp. also had positive results in all 5 individual samples. Thus, we were not able to use clinical samples to confirm the results of the spiked sample study which indicated that culture of a pool would detect shedding even if only 1 of the 5 samples was positive. Additional series of clinical samples are required to determine the usefulness of pooled samples in horses that are intermittent shedders.

Our study indicated that a pool of 5 10‐g aliquots of fecal samples is an acceptable culture sample to accurately determine if a horse is shedding *Salmonella*. Based on the pooling study of spiked samples, a minimum of 10^2^ cfu in 1 of the 5 samples can be detected by standard culture methods. The homogenization protocol is crucial for successful isolation of *Salmonella* from the samples. Typically, feces are collected daily when a *Salmonella* 5‐series sample is being collected. We could find no reason why sampling was spread this far apart. In our study at least 8 hours elapsed between sampling times.

The survival of *Salmonella* spp. in the environment depends, among other things, on the conditions of humidity and temperature of the environment. *Salmonella* spp. has been detected for up to 70 days stored at 25°C.^9^ The survival of *Salmonella* spp. exposed to low temperature (4°C) can last up to 100 weeks.^10^ For that reason, it is reasonable to expect that samples that are not allowed to dry out would remain viable for much longer than 5 days. We did not quantitate the number of bacteria in the clinical samples, and thus we cannot determine if die off of organisms occurred while the samples were waiting to be cultured. Because every horse that was positive on an individual sample was positive with the pooled samples, if a decrease in bacterial numbers occurred it did not appear to affect the results. Evaluating the pattern of positives and negatives in other horses cultured, it would appear that intermittent shedding occurs in horses with *Salmonella*, but continuous shedding is more common.

Our study had several limitations. We only tested horses the clinicians of which elected to perform a *Salmonella* 5‐series, and thus it was a sample limited to high‐risk horses. It is possible that healthy horses without gastrointestinal disease would shed numbers of the organism below the limit of detection. The intervals between sample collection were variable. If it was likely that the horse would be discharged before 5 days, >1 sample was collected each day. Ideally, collection times would be standardized. This variability reflects clinical practice. An additional limitation of the study was that all 5 individual samples that formed the fecal pool tested positive for *Salmonella* spp. in every horse. This result prevented us from testing the hypothesis that fecal pooling would allow identification of *Salmonella* spp. in clinical cases when the bacteria is present in only 1 of the individual samples because of intermittent shedding of the organism. Our study represents a good starting point, but a clinical study with a larger population of horses in which individual and pooled samples can be tested for *Salmonella* spp. still is needed.

In summary, we found that pooling 10‐g aliquots of 5 fecal samples and then culturing the pooled sample was as likely to detect a horse shedding *Salmonella* as submitting 5 individual samples. Because doing so decreased the number of samples submitted to the laboratory by 4, it represents a substantial cost savings. A sensitive and cost‐effective clinical strategy for detecting *Salmonella* in feces from horses would be to submit a pool of 5 fecal samples for *Salmonella* culture.

## CONFLICT OF INTEREST DECLARATION

Authors declare no conflict of interest.

## OFF‐LABEL ANTIMICROBIAL DECLARATION

Authors declare no off‐label use of antimicrobials.

## INSTITUTIONAL ANIMAL CARE AND USE COMMITTEE (IACUC) OR OTHER APPROVAL DECLARATION

Approved by Purdue University IACUC.

## HUMAN ETHICS APPROVAL DECLARATION

Authors declare human ethics approval was not needed for this study.

## Supporting information


**Data S1**: Supporting information
