# Supplementary material for: Recovery of Salmonella bacterial isolates from pooled fecal samples from horses
Source: J Vet Intern Med. 2022 Nov 25;37(1):323–7. doi: 10.1111/jvim.16586 (PMC9889685; doi:10.1111/jvim.16586)
Supplement: Supplementary file 1 — Data S1: Supporting information [file JVIM-37-323-s001.zip › Recovery of Salmonella Bacterial Isolates from Pooled Fecal Samples from....docx]

**Supplemental Information**

**Table 1: Demographics of the horses included in the study**

| Horse Number | Breed | Age (years) | Sex | Days of hospitalization | Presenting Complaint | | Outcome |  |
| --- | --- | --- | --- | --- | --- | --- | --- | --- |
| Horse #1 | Fjord | 14 | Male Castrated | 160 | Colic | Alive | | |
| Horse #2 | Quarter Horse | 1 | Male Castrated | 33 | Eye ulcer | Alive | | |
| Horse #3 | Belgian | 15 | Male Castrated | 9 | Diarrhea | Alive | | |
| Horse #4 | Belgian | 2 | Female | 5 | Colitis | Alive | | |
| Horse #5 | Quarter Horse | 11 | Male Castrated | 5 | Diarrhea, fever | Alive | | |
| Horse #6 | American Saddle Horse | 10 | Male Castrated | 9 | Fever | Alive | | |
| Horse #7 | Thoroughbred | 8 | Female | 6 | Diarrhea, laminitis | Euthanized | | |
| Horse #8 | Welsh Pony | 1 | Male | 5 | Colic | Alive | | |
| Horse #9 | Paint | 6 | Male Castrated | 17 | Diarrhea, fever | Alive | | |
| Horse #10 | Arabian | 13 | Male Castrated | 6 | Colic | Alive | | |
| Horse #11 | Quarter Horse | 11 | Male Castrated | 11 | Sand in the intestines | Alive | | |
| Horse #12 | Paint | 3 | Male Castrated | 2 | Fever | Alive | | |
| Horse #13 | Paint | 18 | Male Castrated | 6 | Colic | Alive | | |
| Horse #14 | Quarter Horse | 3 | Male Castrated | 4 | Colitis | Alive | | |
| Horse #15 | Palomino | 8 | Female | 9 | Colic | Alive | | |
| Horse #16 | Warmblood | 21 | Male Castrated | 28 | Colic | Alive | | |
| Horse #17 | American Saddle Horse | 4 | Female | 54 | Eye trauma | Alive | | |
| Horse #18 | Unknown | 18 | Male Castrated | 6 | Ocular discharge | Alive | | |
| Horse #19 | Unknown | 4 | Female | 8 | Colic | Alive | | |

**Table 2: Results of the *Salmonella* 5-Series Culture and *Salmonella* Pool Culture for each horse included in the study**

| Horse Number | *Salmonella* 5-Series Culture Results | *Salmonella* Pool Culture Results |
| --- | --- | --- |
| Horse #1 | **Positive** | **Positive** |
| Horse #2 | Negative | Negative |
| Horse #3 | Negative | Negative |
| Horse #4 | Negative | Negative |
| Horse #5 | Negative | Negative |
| Horse #6 | Negative | Negative |
| Horse #7 | Negative | Negative |
| Horse #8 | Negative | Negative |
| Horse #9 | **Positive** | **Positive** |
| Horse #10 | **Positive** | **Positive** |
| Horse #11 | Negative | Negative |
| Horse #12 | Negative | Negative |
| Horse #13 | Negative | Negative |
| Horse #14 | Negative | Negative |
| Horse #15 | Negative | Negative |
| Horse #16 | **Positive** | **Positive** |
| Horse #17 | **Positive** | **Positive** |
| Horse #18 | Negative | Negative |
| Horse #19 | Negative | Negative |

| Horse Identification | | | Number of positives | Order of positives | | | | | | |
| --- | --- | --- | --- | --- | --- | --- | --- | --- | --- | --- |
| Horse Number | Age (years) | Presenting Complaint |  | 1 | 2 | | 3 | | 4 | 5 |
| Horse #1 | 3 | Diarrhea | 2 out of 5 | Neg | | Pos | | Pos | Neg | Neg |
| Horse #2 | 11 | Acc Foal | 1 out of 5 | Pos | | Neg | | Neg | Neg | Neg |
| Horse #3 | 4 | Mandibular fracture | 2 out of 5 | Pos | | Pos | | Neg | Neg | Neg |
| Horse #4 | 13 | Colic | 5 out of 5 | Pos | | Pos | | Pos | Pos | Pos |
| Horse #5 | 26 | Anorexia | 5 out of 5 | Pos | | Pos | | Pos | Pos | Pos |
| Horse #6 | 9 | Colic | 5 out of 5 | Pos | | Pos | | Pos | Pos | Pos |
| Horse #7 | 13 | Colic | 5 out of 5 | Pos | | Pos | | Pos | Pos | Pos |
| Horse #8 | 20 | Colic | 1 out of 1 | Pos | |  | |  |  |  |
| Horse #9 | 19 | Colic | 2 out of 5 | Neg | | Pos | | Neg | Pos | Neg |
| Horse #10 | 4 | Eye trauma | 5 out of 5 | Pos | | Pos | | Pos | Pos | Pos |
| Horse #11 | 16 | Colic | 5 out of 5 | Pos | | Pos | | Pos | Pos | Pos |
| Horse #11 (Recheck + 39 days) | 16 | Colic | 2 out of 5 | Neg | | Neg | | Pos | Pos | Neg |
| Horse #11 (Recheck + 54 days) | 16 | Colic | 0 out of 5 | Neg | | Neg | | Neg | Neg | Neg |

**Table 3: Results of the *Salmonella* Culture for horses that presented during the same time period as the subject horses but whose samples were not pooled**

Pos, Positive; Neg, Negative
