# Supplementary material for: Recovery of Salmonella bacterial isolates from pooled fecal samples from horses
Source: J Vet Intern Med. 2022 Nov 25;37(1):323–7. doi: 10.1111/jvim.16586 (PMC9889685; doi:10.1111/jvim.16586)
Supplement: Supplementary file 1 — Data S1: Supporting information [file JVIM-37-323-s001.zip › Recovery of Salmonella Bacterial Isolates from Pooled Fecal Samples from....pdf]

Supplemental Information

Table 1: Demographics of the horses included in the study

Horse Number	Breed	Age (years)	Sex	Days of hospitalization	Presenting Complaint	Outcome
Horse #1	Fjord	14	Male Castrated	160	Colic	Alive
Horse #2	Quarter Horse	1	Male Castrated	33	Eye ulcer	Alive
Horse #3	Belgian	15	Male Castrated	9	Diarrhea	Alive
Horse #4	Belgian	2	Female	5	Colitis	Alive
Horse #5	Quarter Horse	11	Male Castrated	5	Diarrhea, fever	Alive
Horse #6	American Saddle Horse	10	Male Castrated	9	Fever	Alive
Horse #7	Thoroughbred	8	Female	6	Diarrhea, laminitis	Euthanized
Horse #8	Welsh Pony	1	Male	5	Colic	Alive
Horse #9	Paint	6	Male Castrated	17	Diarrhea, fever	Alive
Horse #10	Arabian	13	Male Castrated	6	Colic	Alive
Horse #11	Quarter Horse	11	Male Castrated	11	Sand in the intestines	Alive
Horse #12	Paint	3	Male Castrated	2	Fever	Alive
Horse #13	Paint	18	Male Castrated	6	Colic	Alive
Horse #14	Quarter Horse	3	Male Castrated	4	Colitis	Alive
Horse #15	Palomino	8	Female	9	Colic	Alive
Horse #16	Warmblood	21	Male Castrated	28	Colic	Alive
Horse #17	American Saddle Horse	4	Female	54	Eye trauma	Alive
Horse #18	Unknown	18	Male Castrated	6	Ocular discharge	Alive
Horse #19	Unknown	4	Female	8	Colic	Alive

Table 2: Results of the *Salmonella* 5-Series Culture and *Salmonella* Pool Culture for each horse included in the study

Horse Number	*Salmonella* 5-Series Culture Results	*Salmonella* Pool Culture Results
Horse #1	**Positive**	**Positive**
Horse #2	Negative	Negative
Horse #3	Negative	Negative
Horse #4	Negative	Negative
Horse #5	Negative	Negative
Horse #6	Negative	Negative
Horse #7	Negative	Negative
Horse #8	Negative	Negative
Horse #9	**Positive**	**Positive**
Horse #10	**Positive**	**Positive**
Horse #11	Negative	Negative
Horse #12	Negative	Negative
Horse #13	Negative	Negative
Horse #14	Negative	Negative
Horse #15	Negative	Negative
Horse #16	**Positive**	**Positive**
Horse #17	**Positive**	**Positive**
Horse #18	Negative	Negative
Horse #19	Negative	Negative

Table 3: Results of the *Salmonella* Culture for horses that presented during the same time period as the subject horses but whose samples were not pooled

Horse Identification			Number of positives	Order of positives				
Horse Number	Age (years)	Presenting Complaint		1	2	3	4	5
Horse #1	3	Diarrhea	2 out of 5	Neg	Pos	Pos	Neg	Neg
Horse #2	11	Acc Foal	1 out of 5	Pos	Neg	Neg	Neg	Neg
Horse #3	4	Mandibular fracture	2 out of 5	Pos	Pos	Neg	Neg	Neg
Horse #4	13	Colic	5 out of 5	Pos	Pos	Pos	Pos	Pos
Horse #5	26	Anorexia	5 out of 5	Pos	Pos	Pos	Pos	Pos
Horse #6	9	Colic	5 out of 5	Pos	Pos	Pos	Pos	Pos
Horse #7	13	Colic	5 out of 5	Pos	Pos	Pos	Pos	Pos
Horse #8	20	Colic	1 out of 1	Pos				
Horse #9	19	Colic	2 out of 5	Neg	Pos	Neg	Pos	Neg
Horse #10	4	Eye trauma	5 out of 5	Pos	Pos	Pos	Pos	Pos
Horse #11	16	Colic	5 out of 5	Pos	Pos	Pos	Pos	Pos
Horse #11 (Recheck + 39 days)	16	Colic	2 out of 5	Neg	Neg	Pos	Pos	Neg
Horse #11 (Recheck + 54 days)	16	Colic	0 out of 5	Neg	Neg	Neg	Neg	Neg

Pos, Positive; Neg, Negative

12
